# Differential Inhibition of Human Nav1.2 Resurgent and Persistent Sodium Currents by Cannabidiol and GS967

**DOI:** 10.3390/ijms21072454

**Published:** 2020-04-01

**Authors:** Emily R. Mason, Theodore R. Cummins

**Affiliations:** 1 Department of Pharmacology and Toxicology, Indiana University School of Medicine, IUPUI campus, Indianapolis, IN 46202, USA; 2 Department of Biology, Purdue School of Science, IUPUI campus, Indianapolis, IN 46202, USA; trcummin@iu.edu

**Keywords:** voltage-gated sodium channels, antiepileptic drugs, Nav1.2, CBD, cannabidiol, GS967, Prax330, resurgent current, persistent current

## Abstract

Many epilepsy patients are refractory to conventional antiepileptic drugs. Resurgent and persistent currents can be enhanced by epilepsy mutations in the Nav1.2 channel, but conventional antiepileptic drugs inhibit normal transient currents through these channels, along with aberrant resurgent and persistent currents that are enhanced by Nav1.2 epilepsy mutations. Pharmacotherapies that specifically target aberrant resurgent and/or persistent currents would likely have fewer unwanted side effects and be effective in many patients with refractory epilepsy. This study investigated the effects of cannbidiol (CBD) and GS967 (each at 1 μM) on transient, resurgent, and persistent currents in human embryonic kidney (HEK) cells stably expressing wild-type hNav1.2 channels. We found that CBD preferentially inhibits resurgent currents over transient currents in this paradigm; and that GS967 preferentially inhibits persistent currents over transient currents. Therefore, CBD and GS967 may represent a new class of more targeted and effective antiepileptic drugs.

## 1. Introduction

**Figure 1:**
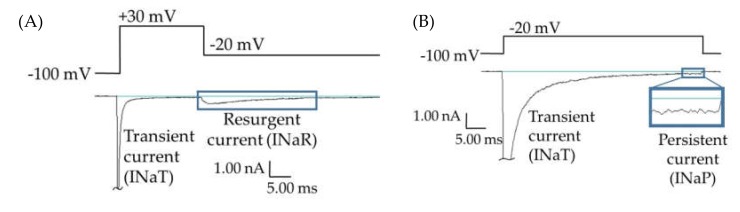
Persistent and resurgent currents. (**A**) A raw current trace from a human embryonic kidney (HEK) cell expressing hNav1.2 cDNA. Peak resurgent current (blue box) was measured as the peak current occurring during the repolarization pulse in the resurgent current voltage protocol (single step from the protocol shown above the trace). (**B**) A raw current trace from an HEK cell expressing hNav1.2 cDNA. Peak persistent current (blue box) was measured as the average current over the last 5 ms of a 50 ms depolarization pulse (shown above the trace). Inset shows persistent current, amplified 5×.

While most epilepsy cases respond well to common antiepileptic drugs, about 38% of epilepsy patients experience persistent seizures under conventional epilepsy treatments [1,2,3,4,5,6]. Many of these refractory epilepsies are believed to be manifestations of mutations in *SCN2A*, the gene for the human voltage-gated sodium channel hNav1.2 [7]. This channel isoform is predominantly expressed in glutamatergic neurons in the brain; and epilepsy mutations in this protein are generally believed to cause a net augmentation of the channel function, leading to hyperexcitability and inappropriate action potential firing. Some of these mutations enhance channel function through the augmentation of resurgent and/or persistent currents (examples of raw data shown in Figure 1) [8,9,10,11,12]. Resurgent currents occur when there is an intracellular blocking molecule present, which binds to the central pore upon depolarization, blocking the transient current and hindering the interaction of the inactivation particle with the central pore. Upon repolarization, the blocking molecule dissociates and current briefly resurges through the central pore before it is again cut off by the inactivation particle (Figure 1A). Persistent current, also called late current, is the current that occurs after most of the Nav channels have opened and inactivated, due to either incomplete inactivation or brief dissociations of the inactivation particle from the central pore (Figure 1B). The enhancement of Nav1.2-mediated persistent and resurgent currents by some *SCN2A* epilepsy mutations is predicted to play a role in epileptogenesis. Thus, these aberrant currents may represent targets in the development of novel antiepileptic drugs (AEDs).

Both persistent and resurgent currents have been shown to occur naturally in mammalian neurons. Resurgent current has been shown to occur in brain regions that have been reported to express Nav1.2, including the globus pallidus [13,14,15], dentate gyrus [14,16,17], and hippocampal CA1 pyramidal neurons [14,16,17,18,19]. The proteins Navβ4 and FGF14, which are both endogenous to mouse cerebellar Purkinje neurons, have been reported to be responsible for generating the endogenous resurgent current seen in these neurons [20,21,22,23]. These proteins are likely also responsible for resurgent currents endogenous to other neuronal subpopulations, and there may be additional molecules endogenous to neurons that act as blocking molecules to induce resurgent currents in neurons [21]. Nav1.6 channels are the primary source of resurgent current in some neuronal populations [24,25]. However, in some rodent neuronal populations, such as cerebellar granule (CG) cell and cerebellar nuclear neurons, Nav1.6 does not seem to be the major isoform generating resurgent currents; in these neurons the Nav1.1 and/or Nav1.2 isoforms have been implicated [26,27,28]. While no study has definitively shown that human neurons expressing Nav1.2 also express resurgent current, recordings from mouse dorsal root ganglion (DRG) neurons have demonstrated that Nav1.2 channels can indeed produce resurgent currents in a neuronal background [29]. Persistent currents have been observed in many populations of neurons in mammalian brains, including subicular neurons from patients with temporal lobe epilepsy [30] and in the soma and proximal processes of healthy adult rat CA1 pyramidal cells [31].

Increases in both resurgent and persistent currents are associated with neuronal hyperexcitability and repetitive firing. Resurgent currents have been identified as drivers of both repetitive action potential activity and spontaneous action potential generation [22,32,33,34,35]. Persistent currents have been shown to support burst firing in neurons from mammalian brains [31,36], and increases in both persistent and resurgent currents through Nav channels have been correlated with increased action potential frequency and burst firing [31,32,33,36,37,38,39,40].

Large persistent currents have been observed in subicular (hippocampal) neurons from human epileptic patients [30], and concurrent enhancements of resurgent and persistent currents have been observed in hippocampal neurons after induction of epilepsy in three rodent models [39,40,41,42]. Several *SCN2A* epilepsy mutations [8,9,10,11,43] have also been shown to increase persistent currents through Nav1.2. Resurgent currents are enhanced by pro-excitatory disease mutations in other voltage-gated sodium channel isoforms which are associated with pain, myotonia congenital, long-QT syndrome, and *SCN8A* epilepsies [35,44,45]. However, the involvement of resurgent currents in the pathogenicity of epilepsy mutations of *SCN2A* has only recently begun to be investigated. Though there is not yet evidence that the enhancement of resurgent current is a primary pathogenic mechanism of *SCN2A* epilepsy mutations, one recent study [12] showed that enhancement of Nav1.2-mediated resurgent currents was among the effects of the R1882Q mutation, which has been reported as likely pathogenic in many epilepsy patients, and which was predicted by Berecki et al. [46] to enhance neuronal excitability. Kearney et al. [47] demonstrated that a mutation that enhances Nav1.2 persistent currents in mouse neurons induces seizures and a strong epileptic phenotype. Although it is not yet clear to what degree Nav1.2-mediated resurgent currents contribute to neuronal excitability, or to what degree neuronal excitability is enhanced by *SCN2A* epilepsy mutations that enhance persistent and/or resurgent currents, it is likely that these atypical sodium currents will enhance excitability where they occur by increasing spontaneous and repetitive firing.

Cannabidiol (CBD), which is currently on the market (as Epidiolex) for the treatment of refractory Dravet Syndrome and Lennox-Gestaut Syndrome [48], has shown promise as an antiepileptic compound in both preclinical and clinical studies [49,50,51,52,53]. Additionally, clinical trials have shown that CBD, alone or as an add-on treatment, reduces seizure frequency in many patients with refractory epilepsy [53,54,55,56,57,58,59]. CBD seems to have multiple antiepileptic mechanisms of action, including antagonism of voltage-gated sodium, potassium, and calcium channels [45,60,61]. CBD has been demonstrated to preferentially inhibit resurgent current over transient current in hNav1.6 channels expressed in human embryonic kidney (HEK) cells, while also blocking the enhancement of persistent current by an epileptogenic hNav1.6 mutation [45]. This preferential inhibition of resurgent and persistent currents has not yet been tested in other neuronal voltage-gated sodium channel isoforms.

One potential AED that is currently in the pharmaceutical pipeline, GS967/Prax330, has demonstrated preferential inhibition of persistent current (I_Na_P) over transient current (I_Na_T) in Nav1.5 and Nav1.6 [62,63], and in tsA201 cells expressing a mutant NaV1.2 cDNA derived from the *Scn2a*Q54 transgene (rat NaV1.2-GAL879-881QQQ) [64]. GS967 has been shown to be antiepileptic in preclinical models of Nav1.1, Nav1.2, and Nav1.6 epilepsies [62,64,65,66]. A phase 1 trial to assess the safety of Prax330 has been completed in Australia, but the results have not yet been reported. The effects of GS967 on persistent currents in cells expressing human Nav1.2 channels have not yet been tested.

Since persistent currents are increasingly being implicated in the pathogenesis of Nav-associated epilepsy, and since resurgent currents may also be a pathogenic effect of Nav1.2 epilepsy mutations, we analyzed of the abilities of CBD and GS967 to selectively block resurgent or persistent current over transient current. Previous studies indicated that CBD can selectively block the resurgent over transient Nav1.6 (but not Nav1.1) current [45] and that GS967 can selectively block persistent over transient Nav1.6 current [62,64]. Since Nav1.6 and Nav1.2 are both predominantly expressed in excitatory neurons in the brain and have similar structures and functions, we predicted that these compounds would express the same selective current inhibition in HEK cells stably expressing wild-type (WT) Nav1.2 channels. The results of our studies support that hypothesis and suggest that, in HEK cells stably expressing WT hNav1.2, CBD preferentially inhibits resurgent current over transient current and GS967 preferentially inhibits persistent current over transient current.

## 2. Results

In order to begin identifying compounds that selectively inhibit persistent (I_Na_P) and resurgent (I_Na_R) sodium currents over transient sodium current (I_Na_T), studies involving two compounds (CBD and GS967) were performed. Epilepsy mutations in both *SCN8A* and *SCN2A* have now been shown to increase I_Na_R. Since we have shown that the enhancement of resurgent current by the *SCN8A*/Nav1.6 epilepsy mutation N1768D can be selectively inhibited by CBD [45], we tested the ability of these compounds to selectively inhibit I_Na_R in the Nav1.2 channel isoform. We performed studies testing the effects of these compounds in cells stably expressing hNav1.2 in the presence of the Navβ4 peptide.

### 2.1. Cannabidiol (CBD) Effects on I_Na_T, I_Na_R, and I_Na_P

**Figure 2:**
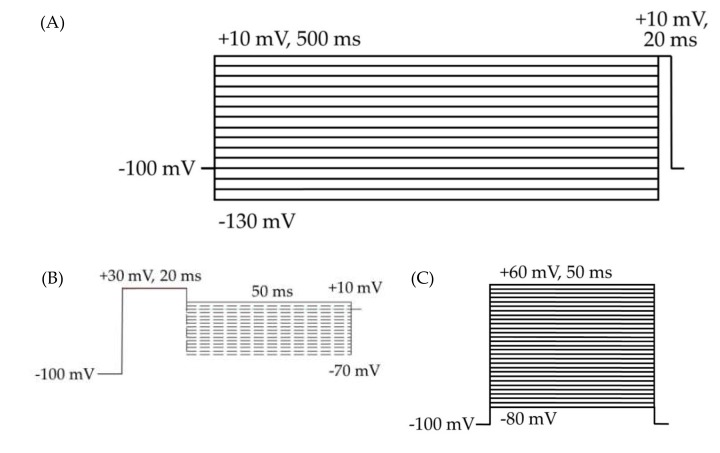
HEK cell electrophysiology voltage protocols. Cells were held at −100 mV for all three protocols. (**A**) Voltage protocol for the analysis of inactivation and transient currents. The 500 ms depolarizing prepulses from −130 to +10 mV, in 10 mV steps, followed by a 20 ms test pulse at +10 mV. (**B**) Resurgent current protocol. (**C**) Voltage protocol for the analysis of activation, transient currents, persistent currents, and gating pore currents. The 50 ms square pulses from −80 to +60 mV, in 5 mV steps.

**Figure 3:**
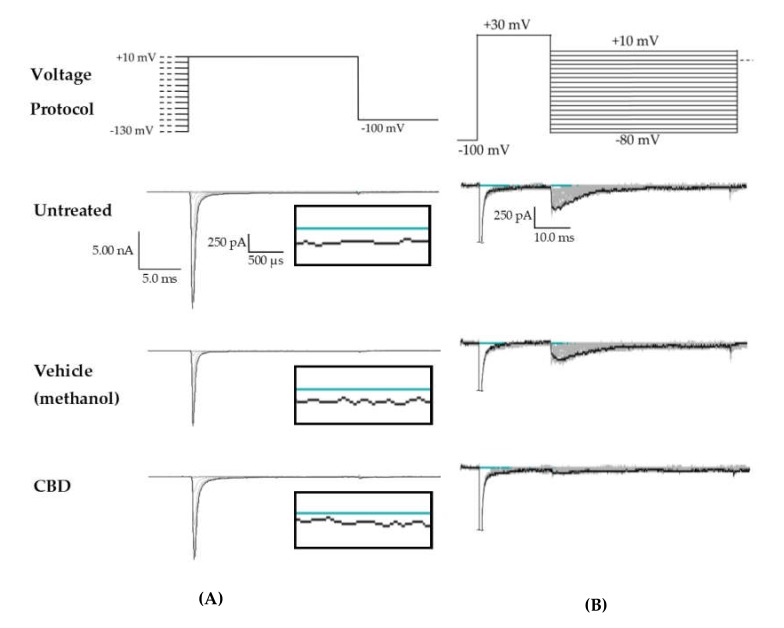
Raw data from cannbidiol (CBD) treatments. (**A**) Voltage protocol used to evaluate transient current (I_Na_T) and persistent current (I_Na_P) (top) and raw current trace families from representative cells, with the trace from the –130 mV prepulse step highlighted. Inset (box) shows zoom view of persistent current (I_Na_P). (**B**) Voltage protocol used to evaluate resurgent current (I_Na_R) (top) and raw current trace families from representative cells, with peak resurgent current trace highlighted.

**Figure 4:**
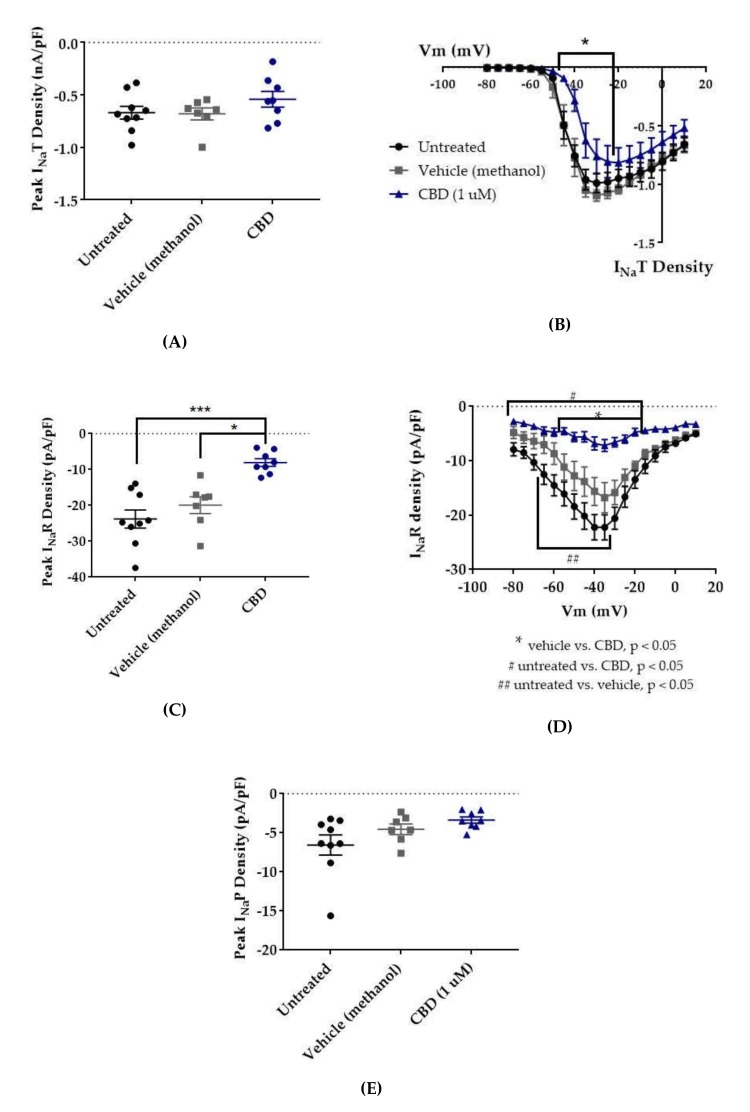
Effects of cannabidiol (CBD) on wild-type (WT) hNav1.2 in stable human embryonic kidney (HEK) cell lines. The Navβ4 peptide was included in the pipette solution for all experiments with CBD. Unless otherwise indicated, * p < 0.05, *** p < 0.001. (**A**) The maximal peak transient current density of each cell is plotted; average and SEM for each group is indicated by bars. Significance was determined by Kruskal–Wallis test. *n* = 9 untreated, 7 vehicle (methanol), 8 CBD. (**B**) The maximal peak resurgent current density of each cell is plotted; average and standard error of the mean (SEM) for each group is indicated by bars. Significance was determined by two-way ANOVA with Tukey’s multiple comparisons. *p < 0.05, CBD vs. vehicle. *n* = 9 untreated, 7 vehicle (methanol), 8 CBD. (**C**) The maximal peak persistent current density of each cell is plotted; average and SEM for each group is indicated by bars. Significance was determined by Kruskal–Wallis test with Dunn’s multiple comparisons. *n* = 9 untreated, 7 vehicle (methanol), 8 CBD. (**D**) Average current densities elicited by voltage steps from −80 to +10 mV. Significance was determined by two-way ANOVA with Tukey’s multiple comparisons. *p < 0.05, CBD vs. vehicle, #*p* < 0.05, CBD vs. untreated, ##*p* < 0.05, vehicle vs. untreated. *n* = 9 untreated, 7 vehicle (methanol), 8 CBD. (**E**) Average peak resurgent current densities over a range of voltages from −65 to +5 mV. Significance was determined by Kruskal–Wallis test. *n* = 9 untreated, 7 vehicle (methanol), 8 CBD.

Since CBD has been shown to inhibit the enhancement of I_Na_R caused by the Nav1.6 epilepsy mutation N1768D without disturbing the I_Na_T density [45], we predicted that CBD would also selectively inhibit I_Na_R and/or I_Na_P over I_Na_T in HEK cells stably expressing WT Nav1.2. Transient current magnitude was measured from the first few steps of the standard inactivation protocol, in which 500-ms prepulses from −130 to −100 preceded a 20-ms test pulse to +10 mV (Figure 2A). Compared to untreated cells, methanol (the vehicle for CBD) had no effect on average maximum peak transient current amplitude (untreated −12.53 ± 1.19 nA, *n* = 9; vehicle (methanol) −12.28 ± 1.31 nA, *n* = 7; Kruskal–Wallis test, *p* = 0.6805) or average maximum peak transient current density (Figure 3A, untreated −0.67 ± 0.06 nA/pF, *n* = 9; vehicle (methanol) −0.68 ± 0.06 nA/pF, *n* = 7; Kruskal–Wallis test, *p* = 0.3591). CBD (1 μM) had no significant effect on average maximum peak transient current amplitude (−10.54 ± 1.24 nA, *n* = 8 CBD, 7 vehicle, 9 untreated; Kruskal–Wallis test *p* = 0.6805) or average maximum peak transient current density (Figure 4A, −0.54 ± 0.07 nA/pF, *n* = 8 CBD, 7 vehicle, 9 untreated; Kruskal–Wallis test, *p* = 0.3591) compared to untreated or methanol-treated cells. However, when the current densities were averaged within groups at each tested membrane potential, CBD significantly inhibited the transient current density, compared to vehicle, from −45 to −35 mV (Figure 4B, vehicle vs. CBD, two-way ANOVA with Tukey’s multiple comparisons, multiple comparisons *p* < 0.05 from −45 to −25 mV; *n* = 7 vehicle, 8 CBD). Therefore, our data suggests that CBD (1 μM) does not significantly reduce maximum peak transient current density, but it does inhibit average current density (compared to vehicle) from −45 to −25 mV in HEK cells stably expressing WT hNav1.2 channels, suggesting that activation is modified (see further analysis below).

Resurgent current densities were measured by a 20-ms depolarization to +20 mV followed by 50-ms respolarization steps from −70 to +10 mV (Figure 2B); the resurgent current occurs during the repolarization phase. Average maximum peak resurgent current densities were unaltered by methanol (Figure 3, Figure 4C, untreated −23.79 ± 2.52 pA/pF, vehicle (methanol) −19.93 ± 2.37 pA/pF, *n* = 9 untreated, 7 vehicle; Kruskal–Wallis test with Dunn’s multiple comparisons, multiple comparisons *p* > 0.9999) but significantly reduced by 1 μM CBD (Figure 3 and Figure 4C, CBD −8.07 ± 1.08 pA/pF; Kruskal–Wallis test with Dunn’s multiple comparisons; CBD vs. vehicle (methanol), *n* = 8 CBD, 7 vehicle; multiple comparisons *p* = 0.0137; CBD vs. untreated, *n* = 8 CBD, 9 untreated; multiple comparisons *p* = 0.0005). When the resurgent current densities were averaged at each tested membrane potential, we observed a slight but significant reduction of average peak resurgent current densities by methanol (Figure 3 and Figure 4D, vehicle (methanol) vs. untreated, *n* = 6 vehicle, 9 untreated; two-way ANOVA with Tukey’s multiple comparisons, multiple comparisons *p* = 0.0533 at −55 mV, *p* = 0.0055–0.0274 at all other measured voltages from −65 to −35 mV) and a further much larger reduction by CBD (Figure 3 and Figure 4D, CBD vs. vehicle (methanol), *n* = 9 CBD, 6 vehicle; two-way ANOVA with Tukey’s multiple comparisons, multiple comparisons *p* = < 0.0001–0.0120 from −55 to −20 mV; CBD vs. untreated, *n* = 9 CBD, 7 untreated; two-way ANOVA with Tukey’s multiple comparisons, multiple comparisons *p* = < 0.0001–0.0305 from -80 to -20 mV). Therefore, resurgent current is moderately reduced by methanol and substantially reduced by 1 μM CBD in HEK cells stably expressing WT hNav1.2 channels.

The peak persistent current density was not significantly reduced by methanol (Figure 3 and Figure 4E, untreated -6.55 ± 1.29 pA/pF, vehicle (methanol) −4.54 ± 0.67 pA/pF, *n* = 9 untreated, 7 vehicle; Kruskal–Wallis test, *p* = 0.0727) and was slightly reduced by CBD (Figure 3 and Figure 4E, CBD −3.38 ± 0.39 pA/pF, *n* = 8 untreated, 7 vehicle, 9 CBD; Kruskal–Wallis test, *p* = 0.0727). Thus, methanol and 1 μM CBD may moderately inhibit persistent current in HEK cells stably expressing WT hNav1.2 channels.

### 2.2. GS967 Effects on I_Na_T, I_Na_R, and I_Na_P

**Figure 5:**
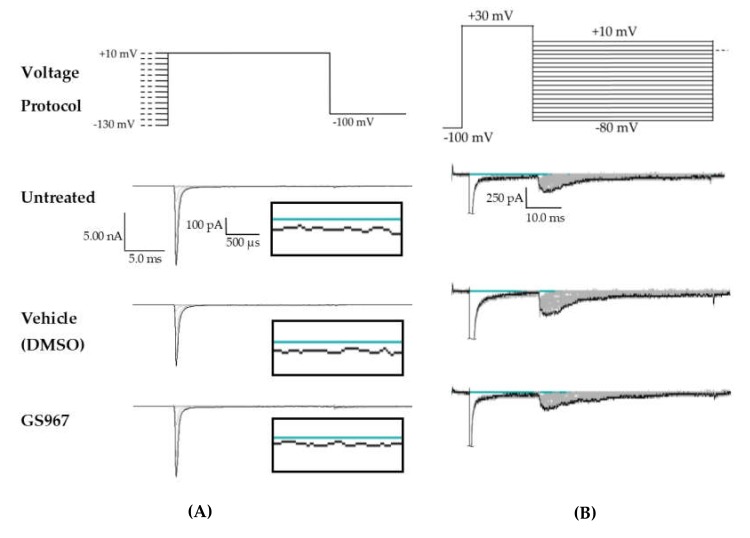
Raw data from GS967 treatments. (**A**) Voltage protocol used to evaluate transient current (I_Na_T) and persistent current (I_Na_P) (top) and raw current trace families from representative cells, with the trace from the –130 mV prepulse step highlighted. Inset (box) shows zoom view of persistent current (I_Na_P). (**B**) Voltage protocol used to evaluate resurgent current (I_Na_R) (top) and raw current trace families from representative cells, with peak resurgent current trace highlighted.

**Figure 6:**
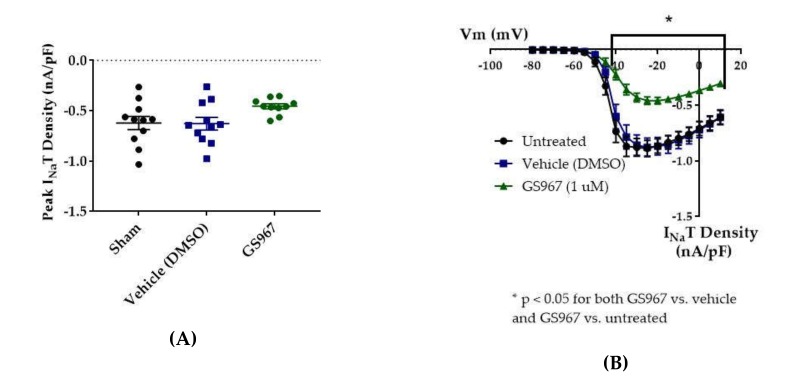

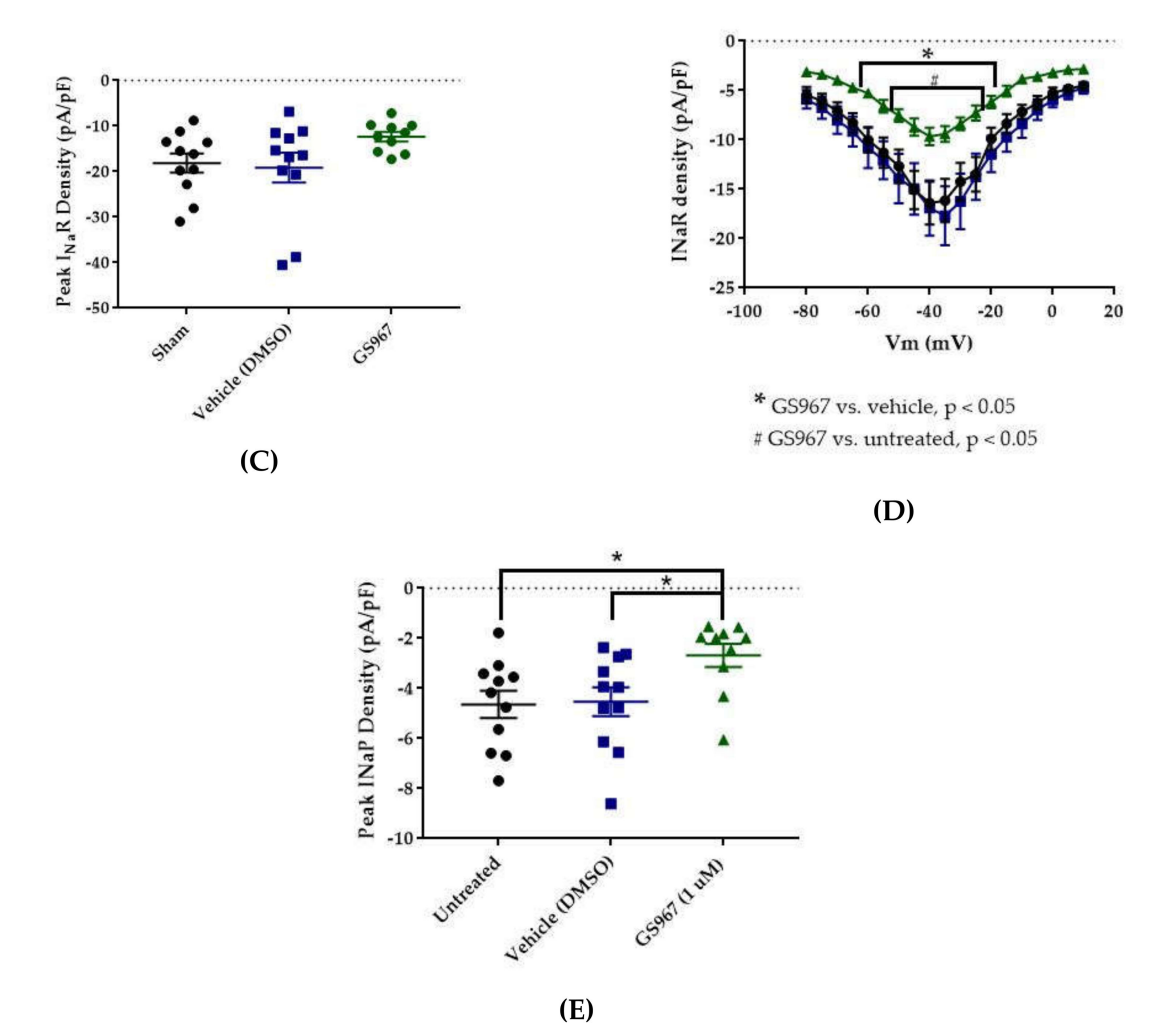
Effects of GS967 on WT hNav1.2 in stable HEK cell lines. The Navβ4 peptide was included in the pipette solution for all experiments with GS967. (**A**) The maximal peak transient current density of each cell is plotted; average and SEM for each group is indicated by bars. Significance was determined by one-way ANOVA. *n* = 11 untreated, 11 vehicle, 10 GS967. (**B**) The maximal peak resurgent current density of each cell is plotted; average and SEM for each group is indicated by bars. Significance was determined by two-way ANOVA with Tukey’s multiple comparisons. *p < 0.05 for both GS967 vs. vehicle and GS967 vs. untreated. *n* = 11 untreated, 11 vehicle, 10 GS967. (**C**) The maximal peak resurgent current density of each cell is plotted; average and SEM for each group is indicated by bars. Significance was determined by one-way ANOVA. *n* = 11 untreated, 11 vehicle, 10 GS967. (**D**) Average current densities elicited by voltage steps from −80 to +10 mV. Significance was determined by two-way ANOVA with Tukey’s multiple comparisons. *p < 0.05, GS967 vs. vehicle, #*p* < 0.05, GS967 vs. untreated. *n* = 11 untreated, 11 vehicle, 9 GS967. (**E**) Average peak resurgent current densities over a range of voltages from −65 to +5 mV. Significance was determined by Kruskal–Wallis test with Dunn’s multiple comparisons. *n* = 11 untreated, 11 vehicle, 10 GS967. DMSO = dimethyl sulfoxide.

Compared to untreated cells, the vehicle (dimethyl sulfoxide, DMSO) had no effect on average maximum peak hNav1.2 transient current amplitude (untreated −11.84 ± 1.31 nA, vehicle (DMSO) −11.33 ± 1.36 nA, *n* = 12 untreated, 11 vehicle; one-way ANOVA *p* = 0.1011) or average maximum peak transient current density (Figure 5 and Figure 6B, untreated −0.62 ± 0.07 nA/pF, vehicle (DMSO) −0.63 ± 0.06 nA/pF, *n* = 11 untreated, 11 vehicle; one-way ANOVA *p* = 0.0685). GS967 (1 μM) slightly reduced average maximum peak transient current amplitude (−8.37 ± 0.47 nA, *n* = 12 untreated, 11 vehicle, 10 GS967, one-way ANOVA *p* = 0.1011) and average maximum peak transient current density (Figure 5 and Figure 6A, −0.46 ± 0.02 nA/pF, *n* = 11 untreated, 11 vehicle, 10 GS967; one-way ANOVA *p* = 0.0685) compared to untreated or methanol-treated cells. However, when the current densities were averaged within groups at each tested membrane potential, GS967 significantly inhibited the transient current density, compared to vehicle, from −40 to +10 mV (Figure 5 and Figure 6B, *n* = 11 vehicle, 10 GS967; two-way ANOVA with Tukey’s multiple comparisons, multiple comparisons *p* = 0.0145 at −40 mV, *p* < 0.0001 from −35 to +5 mV, *p* = 0.0003 at +10 mV). Therefore, our data suggests that GS967 (1 μM) moderately inhibits transient currents in HEK cells stably expressing WT hNav1.2 channels.

Average maximum peak resurgent current densities were unaltered by DMSO (Figure 5 and Figure 6C, untreated −18.16 ± 2.09 pA/pF, vehicle (DMSO) −19.15 ± 3.29 pA/pF, *n* = 11 untreated, 11 vehicle; one-way ANOVA *p* = 0.1193) and only slightly reduced by 1 μM GS967 (Figure 5 and Figure 6C, GS967 −12.35 ± 1.03 pA/pF, *n* = 11 untreated, 11 vehicle, 10 GS967; one-way ANOVA *p* = 0.1193). When the average resurgent current densities were averaged at each tested membrane potential, we observed that DMSO did not alter average peak resurgent current densities (Figure 5 and Figure 6D, vehicle (DMSO) vs. untreated, two-way ANOVA with Tukey’s multiple comparisons, multiple comparisons *p* > 0.5000 at all tested voltages; *n* = 11 vehicle, 11 untreated). Compared to vehicle (DMSO), GS967 significantly inhibited resurgent current densities from −50 to −25 mV (Figure 5 and Figure 6D, GS967 vs. vehicle (DMSO), two-way ANOVA with Tukey’s multiple comparisons, multiple comparisons *p* = 0.0003–0.0326 from −55 to −20 mV; *n* = 9 GS967, 11 vehicle). Therefore, resurgent current is not affected by DMSO and moderately reduced by 1 μM GS967 in HEK cells stably expressing WT hNav1.2 channels.

The peak persistent current density was not significantly reduced by DMSO (Figure 5 and Figure 6E, untreated −4.64 ± 0.54 pA/pF, vehicle (DMSO) −4.53 ± 0.58 pA/pF, *n* = 11 untreated, 11 vehicle; Kruskal–Wallis test with Dunn’s multiple comparisons, multiple comparisons *p* > 0.9999) and was significantly reduced by GS967 (Figure 5 and Figure 6E, GS967 −2.69 ± 0.46 pA/pF, GS967 vs. vehicle (DMSO), Kruskal–Wallis test with Dunn’s multiple comparisons, multiple comparisons *p* = 0.0425; *n* = 10 GS967, 11 vehicle). Thus, while persistent current in HEK cells stably expressing WT hNav1.2 channels is apparently unaffected by DMSO, it is significantly inhibited by 1 μM GS967.

### 2.3. Cannabidiol (CBD) Effects on hNav1.2 Gating

In order to better understand how CBD and GS967 inhibit hNav1.2 currents, we examined the effects of these compounds on the voltage dependence of activation and inactivation.

**Figure 7:**
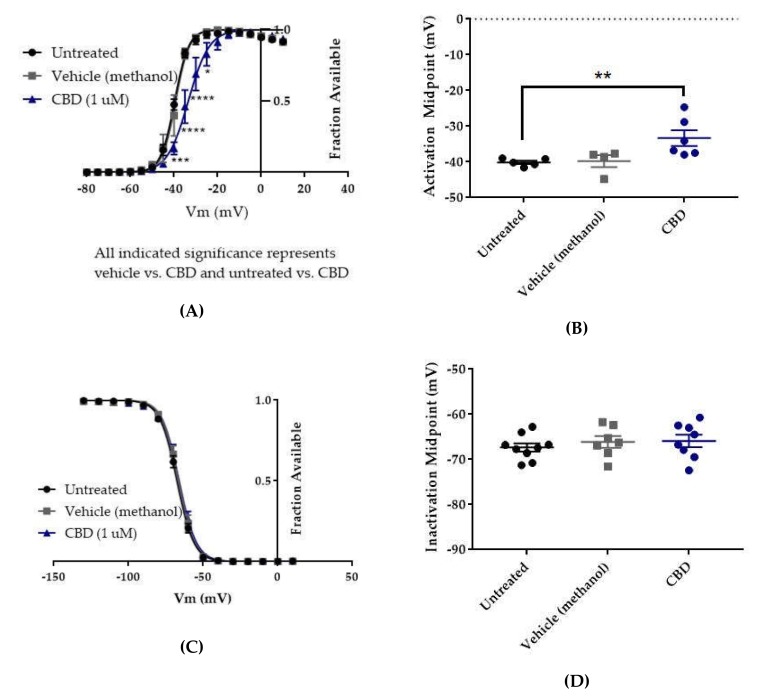
Effects of CBD on WT hNav1.2 gating in stable HEK cell lines. Unless otherwise indicated, * *p* < 0.05, ** *p* < 0.01, *** *p* < 0.001, **** *p* < 0.0001. (**A**) Activation curves. Conductance was calculated as G/Gmax, where G = I/(Vm-Vrev), Vrev = reversal potential, and Gmax = maximum inward conductance across all tested voltages. Significance was determined by two-way ANOVA with Tukey’s multiple comparisons. All indicated significance represents both CBD vs. vehicle and CBD vs. untreated. *n* = 5 untreated, 4 vehicle, 6 CBD. (**B**) The estimated activation midpoint for each cell is plotted; average and SEM for each group is indicated by bars. Significance was determined by Kruskal–Wallis test with Dunn’s multiple comparisons. *n* = 5 untreated, 4 vehicle, 6 CBD. (**C**) Inactivation curves. Fraction available was calculated as I/Imax for each cell at each voltage step. Significance was determined by two-way ANOVA with Tukey’s multiple comparisons. *n* = 9 untreated, 7 vehicle, 8 CBD. (**D**) The estimated inactivation midpoint for each cell is plotted; average and SEM for each group is indicated by bars. Significance was determined by Kruskal–Wallis test. *n* = 9 untreated, 7 vehicle, 8 CBD.

The voltage dependences of activation and inactivation were measured using the standard protocols shown in Figure 2A,B, respectively. The vehicle for CBD, methanol, did not alter the conductance–voltage relationship (Figure 7A, vehicle (methanol) vs. untreated, *n* = 4 vehicle, 5 untreated; two-way ANOVA with Tukey’s multiple comparisons, multiple comparisons *p* = 0.3931 at −40 mV, *p* > 0.9000 at all other tested voltages) or the average estimated midpoint of activation (Figure 7B, untreated −40.14 ± 0.48 mV, vehicle (methanol) −39.78 ± 1.69 mV, *n* = 5 untreated, 4 vehicle; Kruskal–Wallis test with Dunn’s multiple comparisons, multiple comparisons *p* > 0.9999). CBD elicited a depolarizing shift in the voltage dependence of activation, as evidenced by depolarizing shifts in the conductance-voltage relationship (Figure 7A, CBD vs. vehicle (methanol), *n* = 6 CBD, 4 vehicle; two-way ANOVA with Tukey’s multiple comparisons, multiple comparisons *p* = < 0.0001–0.0173 from −40 to −25 mV) and in the average estimated midpoint of activation (Figure 7B, CBD -33.32 ± 2.21 mV, CBD vs. vehicle (methanol), *n* = 6 CBD, 4 vehicle; Kruskal–Wallis test with Dunn’s multiple comparisons, multiple comparisons *p* = 0.1534). Though the shift in the average estimated midpoint of activation was insignificant when compared to the vehicle group, it was significant when compared to the untreated group (Kruskal–Wallis test with Dunn’s multiple comparisons, CBD vs. untreated, *n* = 6 CBD, 5 untreated, multiple comparisons *p* = 0.0069; vehicle (methanol) vs. untreated, *n* = 4 vehicle, 5 untreated, multiple comparisons *p* > 0.9999). Thus, CBD produces a depolarizing shift in the voltage dependence of activation for WT hNav1.2 channels stably expressed in HEK cells.

Neither the vehicle (methanol) nor CBD produced any shift in the voltage dependence of inactivation, as evidenced by the absence of any shift in the inactivation-voltage relationship (Figure 7C, *n* = 9 untreated, 7 vehicle, 8 CBD; two-way ANOVA *p* = 0.4304) or in the average estimated midpoint of inactivation (Figure 7D, untreated -67.34 ± 0.93 mV, vehicle (methanol) -66.11 ± 1.30 mV, CBD -65.9 ± 1.39 mV, *n* = 9 untreated, 7 vehicle, 8 CBD; Kruskal–Wallis test *p* = 0.6159). Thus, CBD does not alter the voltage dependence of inactivation for WT hNav1.2 channels stably expressed in HEK cells.

### 2.4. GS967 Effects on hNav1.2 Gating

**Figure 8:**
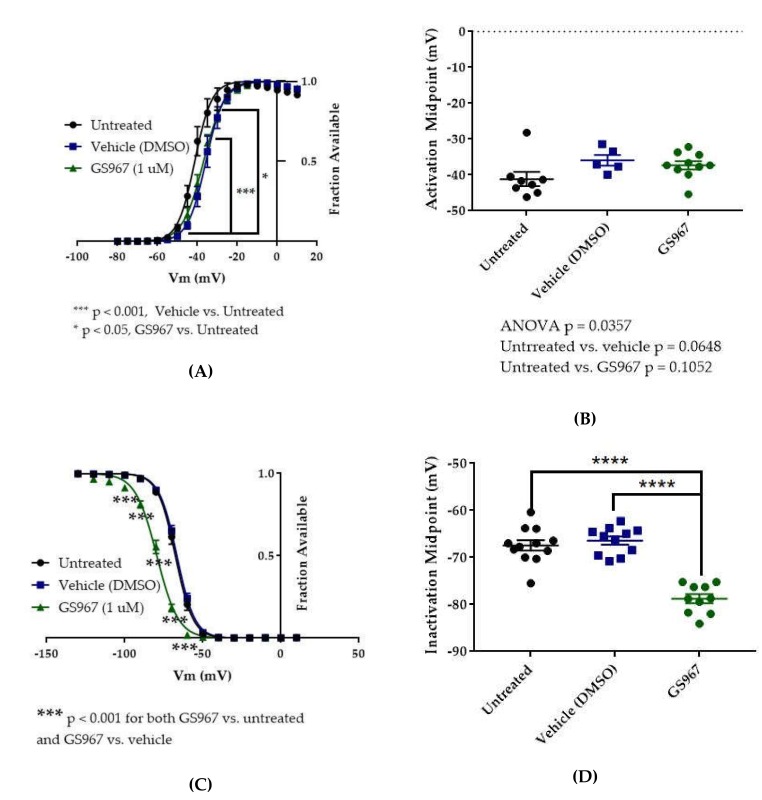
Effects of GS967 on WT hNav1.2 gating in stable HEK cell lines. (**A**) Activation curves. Conductance was calculated as G/Gmax, where G = I/(Vm-Vrev), Vrev = reversal potential, and Gmax = maximum inward conductance across all tested voltages. Significance was determined by two-way ANOVA with Tukey’s multiple comparisons. ***p < 0.0001, vehicle vs. untreated, *p < 0.05, GS967 vs. untreated. *n* = 8 untreated, 5 vehicle, 10 GS967. (**B**) The estimated activation midpoint for each cell is plotted; average and SEM for each group is indicated by bars. Significance was determined by Kruskal–Wallis test with Dunn’s multiple comparisons. *n* = 8 untreated, 5 vehicle, 10 GS967. (**C**) Inactivation curves. Fraction available was calculated as I/Imax for each cell at each voltage step. Significance was determined by two-way ANOVA with Tukey’s multiple comparisons. ***p < 0.001, GS967 vs. untreated and GS967 vs. vehicle. *n* = 12 untreated, 11 vehicle, 10 GS967. (**D**) The estimated inactivation midpoint for each cell is plotted; average and SEM for each group is indicated by bars. Significance was determined by one-way ANOVA with Tukey’s multiple comparisons. *n* = 12 untreated, 11 vehicle, 10 GS967. **** *p* < 0.0001.

The vehicle for GS967, DMSO, significantly depolarized the conductance-voltage relationship (Figure 8A, vehicle (DMSO) vs. untreated, two-way ANOVA with Tukey’s multiple comparisons, multiple comparisons *p* < 0.001 from −45 to −35 mV; *n* = 5 vehicle, 8 untreated) but did not significantly depolarize the average estimated midpoint of activation (Figure 8B, untreated −41.15 ± 1.97 mV, vehicle (DMSO) −35.92 ± 1.53 mV, *n* = 8 untreated, 5 vehicle; Kruskal–Wallis test with Dunn’s multiple comparisons, vehicle (methanol) vs. untreated, multiple comparisons *p* = 0.0648). Compared to vehicle (DMSO), GS967 did not shift the voltage dependence of activation, as evidenced by the absence of any shift in the conductance–voltage relationship (8A, GS967 vs. vehicle (DMSO), two-way ANOVA with Tukey’s multiple comparisons, multiple comparisons *p* = 0.2332–0.8550 from −50 to −35 mV, *p* > 0.9000 at all other tested voltages; *n* = 10 GS967, 5 vehicle) and in the average estimated midpoint of activation (Figure 8B, GS967 −37.30 ± 1.16 mV, Kruskal–Wallis test with Dunn’s multiple comparisons, GS967 vs. vehicle (DMSO), multiple comparisons *p* > 0.9999; *n* = 10 GS967, 5 vehicle). Thus, while DMSO produces a depolarizing shift in the voltage dependence of activation, GS967 has no effect on the voltage dependence of activation for WT hNav1.2 channels stably expressed in HEK cells.

The vehicle (DMSO) did not shift the voltage dependence of inactivation, compared to untreated cells, as evidenced by the absence of any shift in the inactivation-voltage relationship (Figure 8C, two-way ANOVA with Tukey’s multiple comparisons, vehicle (DMSO) vs. untreated, multiple comparisons *p* = 0.1492–0.8390 from −80 to −60 mV, *p* > 0.9000 at all other tested voltages; *n* = 11 vehicle, 12 untreated) or in the average estimated midpoint of inactivation (Figure 8D, untreated −67.41 ± 1.10 mV, vehicle (DMSO) −66.42 ± 0.87 mV, *n* = 12 untreated, 11 vehicle, one-way ANOVA with Tukey’s multiple comparisons, multiple comparisons *p* = 0.7543;). GS967 produced a hyperpolarizing shift in the voltage dependence of inactivation, as evidenced by hyperpolarizing shifts in the inactivation–voltage relationship (Figure 8C, two-way ANOVA with Tukey’s multiple comparisons, GS967 vs. vehicle (DMSO), multiple comparisons *p* < 0.001 from −100 to −60 mV; *n* = 10 GS967, 11 vehicle) and in the average estimated midpoint of inactivation (Figure 8D, GS967 −78.77 ± 0.97 mV, one-way ANOVA with Tukey’s multiple comparisons, GS967 vs. vehicle (DMSO), multiple comparisons *p* < 0.0001; *n* = 10 GS967, 11 vehicle). Thus, GS967 produces a hyperpolarizing shift in the voltage dependence of inactivation for WT hNav1.2 channels stably expressed in HEK cells.

## 3. Discussion

Since mutations in Nav1.2 are generally believed to cause epilepsy through gains of channel function, patients whose epilepsy is reasonably attributable to a *SCN2A* (Nav1.2) mutation are generally expected to respond well to sodium channel blockers, such as carbamazepine, phenytoin, and lamotrigine. Though carbamazepine and high doses of phenytoin have been effective in some *SCN2A* epilepsy patients, many are refractory to these and other conventional antiepileptic drugs (AEDs). These patients are in need of novel, more targeted AEDs that will be effective for them. Even in patients in which conventional sodium channel blockers are effective, they often have intolerable side effects such as fatigue, dizziness, and memory problems. These and other adverse effects of the AEDs sometimes severely decrease the patients’ qualities of life; therefore, there is a general need for new AEDs that can block the mechanism of epileptogenesis without producing devastating adverse effects. These novel, targeted AEDs will likely prove to be efficacious at doses that minimally disturb the healthy conducting activity of the channels, allowing patients to be seizure-free without significant impairment of normal brain activity.

An ideal pharmacotherapeutic strategy for any epileptic patient with a pathogenic *SCN2A* mutation that acts primarily via enhancing resurgent and/or persistent currents may involve selectively inhibiting the aberrantly enhanced resurgent and/or persistent current. Our results suggest that CBD and GS967 preferentially inhibit resurgent and persistent hNav1.2 currents, respectively, over transient hNav1.2 currents, and, thus, that these compounds may have more advantages and be more efficacious than traditional AEDs in the treatment of refractory *SCN2A*-associated epilepsy.

In this study, 1 μM CBD preferentially inhibited resurgent current over transient current in WT hNav1.2. The inhibition of resurgent current density by CBD was significantly different from that of the vehicle and untreated control groups over a wider range of voltages than its inhibition of transient current density. The selectivity of CBD for resurgent current is also suggested by the significant inhibition of maximum peak resurgent current density (see Figure 4C) and the lack of significant inhibition of maximum peak transient current density (see Figure 4A). We also observed that the vehicle for CBD, methanol, mildly reduced hNav1.2 resurgent current densities at negative voltages, but the inhibitory effect of CBD was significant even compared to the methanol effect (see Figure 4C–D). We observed a trend toward inhibition of persistent current by both methanol and CBD (see Figure 4E), though this effect was not significant according to the analysis that we performed. In this study, CBD produced a significant depolarizing shift in the voltage dependence of activation (see Figure 7A,B), which contributed to the inhibition of current densities by limiting channel availability. As seen with WT hNav1.6, 1 μM CBD significantly reduced the maximum resurgent current density in HEK cells stably expressing hNav1.2, while not significantly reducing the maximum peak transient current density (see Figure 4A). These results suggest that, as has been seen in hNav1.6, the selective inhibition of resurgent over persistent currents in hNav1.2 contributes to the proven efficacy of CBD as an AED. Importantly, as CBD does not seem to have the same impact on hNav1.1 resurgent currents [45], it may not impact the excitability of inhibitory neurons.

The same study that demonstrated the ability of CBD to block resurgent currents over transient currents in WT hNav1.6 also demonstrated its ability to selectively block aberrant resurgent and persistent currents generated by epileptogenic *SCN8A* (hNav1.6) mutations [45]. Given the similarities in structure and function between Nav1.2 and Nav1.6, their predominant expression in excitatory (vs. inhibitory) neurons, and the similarities between their responses to CBD, CBD will likely demonstrate selective inhibition of aberrant resurgent and persistent currents caused by epileptogenic hNav1.2 (*SCN2A*) mutations. For example, patients with epilepsy that is driven by *SCN2A* mutations that enhance persistent and/or resurgent current, such as the R1882Q mutation, may benefit from this compound. This mutation depolarizes the voltage dependence of inactivation, slows inactivation, and enhances both persistent and resurgent currents [12]. CBD will likely selectively inhibit the aberrantly enhanced resurgent currents in this mutant form of the hNav1.2 channel. Additionally, if CBD depolarizes the voltage dependence of activation in the mutant channels, as it does in the WT channels, this will compensate for the pathogenic depolarization of the voltage dependence of inactivation in mutant channels by reducing the window current. Both of these effects would dampen or prevent the enhancement of neuronal excitability that is predicted to occur due to this mutation [46], and CBD or other compounds that selectively target persistent currents are thus likely to be beneficial in patients who suffer from otherwise refractory epilepsy attributable to this mutation or other *SCN2A* mutations that enhance persistent and/or resurgent currents.

The results of our study suggest that GS967 preferentially inhibits persistent current over transient current in hNav1.2. This preferential inhibition is supported by the fact that the compound inhibited maximum persistent current density significantly (see Figure 6E), while its effect on maximum transient current density was statistically insignificant (see Figure 6A). Furthermore, the average maximum peak persistent current density was inhibited to a greater degree (40.6% inhibition) than the average maximum peak transient current density (27% inhibition). In this study, GS967 inhibited resurgent current densities to a similar degree as transient densities (see Figure 6A–D). The significant inhibition of transient and resurgent current densities in the current density–voltage relationships can be attributed to the depolarizing shift in the voltage dependence of activation elicited by DMSO and the hyperpolarizing shift in the voltage dependence of inactivation elicited by GS967, which both reduce channel availability. These results are congruent with previous reports that GS967 selectively inhibits persistent current over transient current and hyperpolarizes the voltage dependence of inactivation in pyramidal neurons from the brains of mouse models of *SCN8A* epilepsy and in ND7/23 cells expressing the corresponding mutation [62,63,66]. Additionally, as in this study, WT Nav1.6 resurgent current was not significantly inhibited by GS967 in pyramidal subiculum neurons from one of these mouse models, though the compound did inhibit resurgent and persistent currents that were aberrantly enhanced by the epilepsy mutation [63]. If the GS967 preferentially inhibits persistent currents over transient currents in mutant hNav1.2 channels, as is suggested by its effects on the WT channels, GS967 would be predicted to counteract the enhancement of neuronal excitability that is presumably caused by the epilepsy mutations. Thus, GS967 may prove to be beneficial in patients with refractory epilepsy linked to *SCN2A* mutations that enhance persistent currents, such as the patients with the R1882Q mutation.

Investigation into the effects of enhancements of Nav1.2-mediated resurgent current on neuronal excitability have just begun and are thus not yet firmly established. Resurgent currents have been shown to be enhanced by the R1882Q mutation, which is one of the most frequently reported epilepsy mutations in the *SCN2A* gene, and future studies may reveal the enhancement of resurgent currents to be a common effect of *SCN2A* epilepsy mutations. Patients with this and any other *SCN2A* mutations that enhance resurgent hNav1.2 currents would likely benefit from CBD (Epidiolex), which preferentially inhibits resurgent currents over transient currents in hNav1.2. Enhancements of Nav1.2-mediated persistent currents have been identified as resulting from six of 23 *SCN2A* epilepsy mutations that have been studied in vitro. Two of these mutations, A263V and R1882Q, are among the most frequently reported epilepsy mutations in the *SCN2A* gene and are implicated in epilepsy cases that are refractory to conventional antiepileptic drugs. Therefore, though only 26% of the characterized *SCN2A* epilepsy mutations enhance persistent current, these mutations are presumably responsible for many cases of refractory epilepsy in which a more targeted antiepileptic pharmacotherapy that preferentially inhibits the aberrantly enhanced persistent currents might be highly valuable. This study suggests that GS967 may represent such a drug.

Though Nav1.6 is believed to be the predominant mediator of endogenous resurgent current in central nervous system neurons, aberrant enhancements of Nav1.2-mediated persistent and/or resurgent current by *SCN2A* epilepsy mutations may augment the overall voltage-gated sodium influx in affected neurons and thus increase their excitability. Thus, preferentially inhibiting resurgent and/or persistent currents in patients with *SCN2A* epilepsy mutations that enhance one or both of these currents may prove to be therapeutically beneficial for these patients. This study is the first to investigate the effects of CBD on persistent and resurgent currents in Nav1.2, and it is the first to investigate the effects of GS967 on the function of any naturally occurring variant of Nav1.2. The effects of CBD on Nav1.2 channels bearing epilepsy mutations have not yet been studied.

Given that CBD and GS967 preferentially target resurgent and persistent currents over transient currents in Nav1.2, these compounds could prove to be more effective AEDs than standard treatments such as phenytoin for patients with epilepsies presumably caused by *SCN2A* mutations that enhance one or both of these currents, such as the A263V or R1882Q mutations. Indeed, CBD has already been proven reasonably safe, tolerable, and effective in human patients with epilepsy that is refractory to conventional AEDs [52,53,54,55,56,57,58,59]. Though GS967 has been shown to be an efficacious antiepileptic in rodent models of *SCN1A*, *SCN2A*, and *SCN8A* epilepsies [63,64,65,66], the safety, tolerability, and antiepileptic efficacy of GS967/Prax330 in human patients with refractory epilepsy remains to be fully investigated.

Ultimately, we expect that this research will contribute to a knowledge base that will lead to more effective treatments for patients with epileptogenic *SCN2A* mutations. The results of this and previous studies suggest that CBD and GS967 selectively inhibit, respectively, resurgent and persistent currents over transient currents in Nav1.2, as well as in Nav1.6. Therefore, these compounds may be among the first in a novel subclass of AEDs that selectively target currents aberrantly enhanced by epileptogenic voltage-gated sodium channel mutations.

## 4. Materials and Methods

### 4.1. DNA Constructs

Codon-optimized human WT Nav1.2 DNA constructs were designed in-house and synthesized by GenScript (Piscataway, NJ, USA). The amino acid sequence for the synthesized wild-type hNav1.2 cDNA construct corresponds with NG_008143.1 in the NCBI database. The *SCN2A* gene is contained within a pcDNA3.1 vector and preceded by a CMV promoter.

### 4.2. HEK293 Cell Culture

The use of HEK293T cells (hereafter referred to as HEK cells) [67] was approved by the Institutional Biosafety Committee and followed the ethical guidelines for the National Institutes of Health for the use of human-derived cell lines. HEK cells were grown under standard tissue culture conditions (5% CO2, 37 °C) in maintenance media consisting of DMEM supplemented with 10% fetal bovine serum and 1% penicillin/streptomycin.

### 4.3. Generation of Stable Cell Lines

Stable cell lines were generated by transfecting cells with the wild-type or mutant channel construct, which contains a gene conferring resistance to geneticin (G418). The calcium phosphate precipitation method of transfection was used. Briefly, calcium phosphate-DNA mixture (2.5 μg channel construct) was added to cells in serum-free media for 8–17 h, after which the cells were washed with maintenance media and the serum-free media was replaced with maintenance media. Transfected cells were identified by presence of fine particulate coating the cells before washing. Then, 48 h post-transfection, the cells were split into a 100 mm dish and G418 (500 μg/mL) was added to the media. Media, including G418, was replaced 48 h later. Once cell colonies were visible to the naked eye, colonies were picked and re-plated individually as independent stable cell lines. Stable cell lines were maintained in maintenance media (1X DMEM, 10% FBS, 1% penicillin/streptomycin) containing 500 μg/mL G418.

### 4.4. HEK Electrophysiology

Currents were measured at room temperature (≈22 °C) using a HEKA EPC 10 amplifier and the Pulse software (v8.80, HEKA Elektronik GmbH, Ludwigshafen am Rhein, Germany) for data acquisition. Electrodes were fabricated from 100 μL calibrated pipettes (Drummond Scientific Company, Broomall, PA, USA; cat. # 2-000-100; capillary glass) and fire-polished to a resistance of 1.0–2.0 MΩ using a *p*-1000 micropipette puller (Sutter Instrument Company, Novato, CA, USA). For each cell, a GΩ seal was obtained before breaking into the whole-cell configuration. Voltage protocols were initiated 5 min after entering the whole-cell configuration, allowing time for each cell’s cytoplasm to equilibrate with the pipette solution, while also controlling for time-dependent shifts in sodium channel properties. Voltage errors were minimized by using series resistance compensation (up to 90%), and passive leak currents were cancelled by *p*/-5 subtraction. The bath solution contained (in mM): 140 NaCl, 3 KCl, 1 MgCl_2_, 1 CaCl_2_, and 10 HEPES, adjusted to pH 7.30 with NaOH. The pipette solution contained (in mM): 130 CsF, 10 NaCl, 10 HEPES, and 1 CsEGTA, adjusted to pH 7.30 with CsOH. Navβ accessory subunits were not co-transfected with the Nav1.2α subunit, which functions independently as a channel, due to the variability that this would introduce into the experiments and the lack of information regarding which Navβ subunits are associated with the Nav1.2α subunits in the affected neurons in the brain. The Navβ4 peptide (KKLITFILKKTREK-OH, used at 200 μM) (Biopeptide Co., Inc., San Diego, CA, USA), which corresponds to part of the C-terminal tail of the full-length Nav β4 subunit, was included in the pipette solution in order to induce the resurgent currents. This peptide has been shown to induce resurgent currents in HEK cells expressing voltage-gated sodium channels, while, for unknown reasons, the full-length Navβ4 peptide is not sufficient to produce resurgent currents in HEK cells expressing voltage-gated sodium channels [68,69].

### 4.5. HEK Voltage Protocols

All HEK cells were held at -100 mV.

Measures pertaining to voltage-dependent activation were taken from a voltage protocol consisting of 50 ms test pulses to voltages from -80 to +60 mV, in 5 mV steps (Figure 2C). Inactivation was measured by a 500 ms prepulse step to voltages from -130 to +10 mV, in 10 mV steps, followed by a 20 ms test pulse to +10 mV (Figure 2A). Measurements of transient and persistent currents were derived from the first several sweeps in the inactivation protocol, which consisted of 500 ms hyperpolarizing pulses from -130 to +10 mV followed by a 20 ms depolarizing test pulse to +10 mV (Figure 2A). Persistent currents were measured, in Pulsefit (v8.80, HEKA Elektronik GmbH, Ludwigshafen am Rhein, Germany), as an average of the current amplitude over the last 10% of the test pulse of the inactivation protocol (Figure 2A), from 18 to 20 ms. Resurgent currents were elicited by a 20-ms pulse to +30 mV, followed by a 50-ms repolarization step of voltages from +10 to -65 mV (Figure 2B). The resurgent current peak amplitude was measured, in Pulsefit, as the average minimum value of the current elicited during three sweeps at each repolarization voltage.

Peak current amplitudes, for each voltage, were measured as the minimum value of the current over the entirety of the test pulse. Current densities were calculated by dividing raw current amplitudes by the membrane capacitance value of each cell. Maximal current densities were calculated as the maximal peak current density, at any voltage, for each particular cell. Average current densities represent the average current density at each particular tested voltage.

The reversal potential was estimated, for each cell, by extrapolation of the ascending limb of the current-voltage (IV) curve. The conductance values were then calculated and normalized within each cell.

### 4.6. Drug Application

HEK293 cells stably expressing WT hNav1.2 channels were cultured in maintenance media (DMEM with 10% FBS and 1% penicillin/streptomycin) containing G418 (500 μg/mL). Cells were used 2 days (36–52 h) after splitting. The cells to be studied were preincubated at 37 °C for 15 min in either plain DMEM (untreated controls) or DMEM containing the pharmacological treatment (vehicle or vehicle + compound). Following the preincubation, the DMEM media was removed and replaced with HEK bath solution (recipe provided in Section 4.4). Vehicle and compound treatments were added to the bath solution for those groups, at the same concentrations as in the preincubation.

Tested compounds included cannabidiol (CBD, Cayman Chemicals, Ann Arbor, MI, USA) and GS967 (Cayman Chemicals, Ann Arbor, MI, USA). Cannabidiol was dissolved in methanol at a stock concentration of 1 mM and stored at -20 °C. GS967 was dissolved in dimethyl sulfoxide (DMSO) at a stock concentration of 1 mM and stored at -20 °C. Both compounds were used at 1 μM final concentrations. A concentration of 1 μM was chosen with each compound in order to aid comparisons with previous studies that used this concentration with Nav1.5 (GS967; [70]) and with Nav1.6 (CBD; [45]) currents. Stock solutions were stored as 2 μL aliquots, one of which was diluted in each DMEM preincubation (2 mL) and each bath solution (2 mL) for each 35 mm dish of cells, yielding a final solution comprised of 0.1% vehicle or compound solution.

### 4.7. Statistics and Analysis

Electrophysiological data were analyzed using Pulsefit (v.8.80, HEKA Elektronik GmbH, Ludwigshafen am Rhein, Germany), Microsoft Excel, Origin (v9.1, OriginLab Corp., Northampton, MA, USA), and GraphPad Prism (v.7.03, GraphPad Software, Inc., San Diego, CA, USA). All data points are presented as mean ± standard error of the mean (SEM), and *n* is the number of cells from which contributing data was collected. Current density and conductance were calculated as previously described (see Section 4.5), [71]. Activation and inactivation midpoints were estimated by fitting the current-voltage (IV) plot and inactivation curve for each cell to a current-voltage equation and the Boltzmann equation, respectively, in Pulsefit. Inactivation time constants were estimated by fitting the decay portion of each sodium current trace to a single-exponential equation in Pulsefit.

The normality of data distribution was evaluated with the D’Agostino and Pearson normality test. If the data was normally distributed, a parametric test was used; if the data was not normally distributed, a nonparametric test was used. For single-measure comparisons between all three treatment groups, one-way ANOVAs were used. If the data was normally distributed, an ordinary one-way ANOVA with Tukey’s multiple comparisons test was used; if the data was not normally distributed, the nonparametric Kruskal–Wallis with Dunn’s multiple comparisons test was used. For data comparisons spanning multiple voltages, a two-way ANOVA with Tukey’s multiple comparisons test was used. Statistical significance was set at α = 0.05. When only the multiple comparisons test *p* value is reported for an ANOVA, it is implied that a significant *p* value (*p* < 0.05) was obtained in the ANOVA test. For all figures, unless otherwise specified, * *p* < 0.05, ** *p* < 0.01, *** *p* < 0.001, and **** *p* < 0.0001.

## Abbreviations

AED	Antiepileptic Drug
CBD	Cannabidiol
DMSO	Dimethyl Sulfoxide
HEK	Human Embryonic Kidney (cells)
I_Na_P	Persistent Sodium Current
I_Na_R	Resurgent Sodium Current
I_Na_T	Transient Sodium Current
Nav	Voltage-Gated Sodium (channel)
WT	Wild-Type
